# The State of School Infrastructure in the Assembly Constituencies of Rural India: Analysis of 11 Census Indicators from Pre-primary to Higher Education

**DOI:** 10.3390/ijerph17010296

**Published:** 2020-01-01

**Authors:** Akshay Swaminathan, Menaka Narayanan, Jeff Blossom, R. Venkataramanan, Sujata Saunik, Rockli Kim, S.V. Subramanian

**Affiliations:** 1 Quantitative Sciences, Flatiron Health, New York, NY 10013, USA; akshay325@gmail.com; 2 Palantir Technologies, New York, NY 10014, USA; menaka.narayanan@gmail.com; 3 Center for Geographic Analysis, Harvard University, Cambridge, MA 02138, USA; jblossom@cga.harvard.edu; 4 Warwick Manufacturing Group (WMG), University of Warwick, Coventry CV4 7AL, UK; venkat.ramachandran@gmail.com; 5 Additional Chief Secretary, Skill Development and Entrepreneurship Department, Mumbai 400021, India; ssaunik@gmail.com; 6 Division of Health Policy and Management, College of Health Sciences, Korea University, Seoul 02841, Korea; 7 Department of Public Health Sciences, Graduate School, Korea University, Seoul 02841, Korea; 8 Harvard Center for Population & Development Studies, Cambridge, MA 02138, USA; 9 Department of Social and Behavioral Sciences, Harvard T.H. Chan School of Public Health, Boston, MA 02115, USA

**Keywords:** India, assembly constituencies, geopolitical units, GIS, census, education infrastructures, social determinants of health

## Abstract

In India, assembly constituencies (ACs), represented by elected officials, are the primary geopolitical units for state-level policy development. However, data on social indicators are traditionally reported and analyzed at the district level, and are rarely available for ACs. Here, we combine village-level data from the 2011 Indian Census and AC shapefiles to systematically derive AC-level estimates for the first time. We apply this methodology to describe the distribution of 11 education infrastructures—ranging from pre-primary school to senior secondary school—across rural villages in 3773 ACs. We found high variability in access to higher education infrastructures and low variability in access to lower education variables. For 40.3% (25th percentile) to 79.7% (75th percentile) of villages in an AC, the nearest government senior secondary school was >5 km away, whereas the nearest government primary school was >5 km away in just 0% (25th percentile) to 1.9% (75th percentile) of villages in an AC. The states of Manipur, Arunachal Pradesh, and Bihar showed the greatest within-state variation in access to education infrastructures. We present a novel analysis of access to education infrastructures to inform AC-level policy and demonstrate how geospatial and Census data can be leveraged to derive AC-level estimates for any population health and development indicators collected in the Census at the village level.

## 1. Introduction

Collecting data on indicators of population health and social development can be an effective way to maintain accountability in government [1]. Consistent measurement of such data can foster evidence-based developmental interventions, data-driven resource allocation, and increased accountability for local administrative leaders and policymakers. In India, population health studies and surveys have historically been conducted and reported at the district and state levels [2].

However, recent studies have suggested that geopolitical units with elected representatives may be more relevant units of analysis for population indicator studies than districts, which do not have elected representatives in national government [3,4]. Two such geopolitical units are parliamentary constituencies (PCs) and assembly constituencies (ACs) in India. Providing data at the PC and AC levels can be more useful for policymakers than providing district-level estimates since PC and AC representatives receive government resources to carry out development projects specifically targeting their constituents. While recent studies have provided methods for generating PC-level estimates [3,4], no attempt has been made to produce a dataset that allows analysis at the AC-level.

### 1.1. Assembly Constituency

Countrywide, there are 4120 ACs represented by members of the legislative assembly (MLAs). MLAs are elected to the legislature of their state governments and serve five-year terms. Similar to members of Parliament (MPs) at the PC level who hold positions in the national Parliament and focus on developing national legislature, MLAs are appointed to their state legislature and focus on state issues [5]. This state-specific focus gives MLAs a valuable perspective on local issues that fall outside the purview of the central government [5]. MLAs are provided with resources to enact local development initiatives in their ACs. The MLA Constituency Development Scheme is a state-funded mechanism through which MLAs can receive funds to implement policies that benefit their constituents [6]. MLAs have exclusive autonomy in controlling these funds, which on one hand is directly empowering, but on the other may leave development funds open to corruption and electioneering [7]. State legislatures have Public Accounts Committees (PACs) that make policy recommendations and streamline expenditures of state politicians. Compiling data at the AC-level can help PACs inform local interventions and provide timely progress indicators to guide local politicians. However, to date, there has been no systematic collection of AC-level population development indicators.

### 1.2. Education Infrastructure

Countrywide access to education has been a key goal of the Indian government since its inception. The first Census after independence found that only 9% of women and 27% of men were literate [8]. This spurred the government into setting a goal to provide free compulsory education to all children under 14 by year 1960 [8]. Decades of ensuing governmental action boosted literacy rates—in 2011, the nationwide literacy rate was 65.5% for women and 82.1% for men (58.8% for women and 78.6% for men in rural India) [9]. Beyond literacy rates, these efforts to increase school enrollment brought about long term payoffs for human capital development and population health. Investment in rural education has significantly affected human capital growth, expanding nonagricultural employment and increasing wages [10]. Primary and middle schools boast the highest enrollment rates in the Indian education system—in the 6–14 age group, enrollment has been above 96% since 2010. However, this attendance rate varies by state—based on random visits to primary and middle schools in various states, five states showed attendance levels higher than 85%, while five states showed attendance rates lower than 60% [11]. As of 2018, 30.9% of these school-going children ages 6–14 attended private schools [11]. Enrollment drops as children age—in 2016, secondary schools saw a gross enrollment ratio (the ratio of the number of students to those who qualify for the grade level) of 75.18% [12]. On the opposite end of the spectrum, pre-primary education has seen growth in recent years—in 2018, 66.7% of 3-year-olds were enrolled in some form of a preschool program [11].

Access to education at every level–from early childhood to adolescence–can influence human capital achievement. Over the past few decades, more attention has been drawn to the substantial number of children from low-income households who enter primary school unprepared and already lagging behind their peers. This has galvanized stronger government involvement in pre-primary education [13]. One key early-education government initiative is the Integrated Child Development Scheme (ICDS), a preschool intervention for children ages 0–6 years old in India. Launched in 1975, the program uses ICDS centers (also called Anganwadi centers) to administer interventions like nutrition programs, immunizations, health checkups, nutritional education for mothers, and preschool education for children. In Anganwadi centers, preschool education involves physical activities for children, basic health education, and instruction of topics like alphabets, numbers, and rhymes [14]. A strong pre-primary education sets up children for stronger performance in primary school.

Economic returns of primary education in India are well-studied—for example, one study using data from Madhya Pradesh and Tamil Nadu showed that with each additional year of primary or middle school, women’s wages increased by about 10% and men’s wages by about 8% [15]. Primary education is also important as a prerequisite for other avenues of success, unlocking access to secondary and university education [15]. Secondary school education has been shown to be a gateway to more competitive opportunities in the labor market and may therefore be more effective in raising children out of intergenerational poverty [16]. Over the past few decades, enrollment in private schools has been on the rise even among students from disadvantaged backgrounds. In a 2003 survey of rural primary schools, 28% of the rural population were found to have access to private schools in their own village. 20% of students in these private schools were first generation learners [17].

The availability of physical school infrastructure is of key importance for improving educational outcomes like enrollment and attendance—a trend visible worldwide. A long commute to school has been shown to increase fatigue and rates of attrition amongst school children [18]. Geographically targeted provision of schools can improve access and reduce inequities in the education system [19]. One relevant study analyzed a program in Peru that financed several microprojects involving building and reconstructing schools. The study concluded that increased government spending on school construction improved attendance, especially among poor children [20]. Another study of a 1974 Indonesian government program that built 61,000 schools throughout the country showed increased primary school enrollment as well as an increase in earnings over time [21]. In India, the District Primary Education Program (1995–1999) sought educational reform at the district level through constructing primary schools, enrolling students, training teachers, and seeking participation from the community. Districts that participated in this program saw significantly higher learning achievement and increased primary school enrollment by 5.5–6% [22].

School achievement is not simply a function of infrastructure. Economic circumstances and demographics of a family can often affect how likely the children are to enroll in and succeed at school. For example, female children are less likely than male children to travel long distances to school, due to safety concerns of many parents [18]. Additionally, a lack of effort from the school administration can be deterring—by not adhering to timetables, grouping children from multiple grades together in classrooms, or not providing textbooks and other school supplies, schools can hinder student learning through sheer disorganization. Improperly trained teachers and poorly designed curriculum can also have adverse effects on student achievement [23].

### 1.3. Objective

Given the direct power of MLAs to construct and maintain schools, paired with the availability of Census data on local infrastructure, we choose in this analysis to focus on education infrastructure. We conduct a detailed analysis of the distribution of various education infrastructures at the AC level in rural India. Our methodology leverages three disparate datasets to generate AC-level estimates, thus optimizing the utility of available data for policymakers and researchers interested in ACs. Specifically, we link village-level Census data to corresponding ACs by joining the village border shapefile with the AC border shapefile, allowing for the estimation of AC performance on various Census demographic and amenities indicators. We apply this methodology to analyze and rank ACs by their level of access to 11 education infrastructures of both private and public schools ranging from pre-primary to senior secondary education. Ensuring accessibility of education infrastructure is key to making the Indian education system fair towards all children [18]. The AC unit specifically is important to education facilities because MLAs are held responsible for the construction and maintenance of education infrastructure at a local level. For instance, in Tamil Nadu, as of 2011, MLAs received Rs. 2 crore to address “critical infrastructure gaps” in their ACs. Of these annually allocated funds, Rs. 1.125 crore was earmarked for priority infrastructure, including facilities for schools and construction of Anganwadi centers [6]. Given this context, AC-level estimates can aid MLAs in implementing local policy to promote accessibility to education infrastructure for their constituents.

## 2. Materials and Methods

### 2.1. Data Sources

We used three sources of data for our analysis. The first was the Village Amenities data from the 2011 Indian Census District Census Handbook website of the Office of the Registrar General & Census Commissioner, Government of India (http://www.censusindia.gov.in/2011census/dchb/ DCHB.html). This dataset was restricted to rural India and did not include urban areas. Of the 640,948 villages with amenities features (e.g., availability of schools, health centers, water sources), we used in our analysis the 597,618 that were inhabited (had a nonzero population). From this dataset, we used 11 amenities features that represented village-level availability of education infrastructure.

The second source of data was the 2011 Census Village Boundary and Demographics data published by ML Infomap and purchased by Harvard in 2016 [24]. This dataset was accessed from the Harvard Geospatial Library (http://hgl.harvard.edu:8080/opengeoportal/) by the Harvard Center for Geographic Analysis. To map villages, ML Infomap collected individual taluka/tehsil paper maps and, where possible, small-scale Census Atlas maps from the Registrar General of India. These maps were scanned to vectorize boundaries of villages as polylines and locations of village settlements as points [24]. Then, geographic coordinates were sourced from high resolution satellite images and transferred onto the digitized maps using visibly identifying features such as roads, railway, or water shared between both maps [24]. All villages were linked to the 2011 Census demographics (e.g., total population, proportion of scheduled castes, literacy rate) by ML Infomap. The dataset contained point shapefiles representing village locations for 14 states, and polygon shapefiles representing village boundaries for 22 states. The polygonal village locations were converted to point locations using the ArcGIS “Features to Point” function, which generates a point with latitude and longitude values in the geographic center of a polygon. After this process, there were in total 654,153 units (villages plus urban “towns”) mapped as point locations. Since the Village Amenities dataset was restricted to rural villages, we excluded 8109 urban units (towns), as well as 48,418 units with zero population from this file. This resulted in a total of 597,626 inhabited rural villages. We ascribed geographic metadata to these villages using subdistrict (tehsil) boundaries provided by ML Infomap and state and district boundaries downloaded from the Database of Global Administrative Areas (https://gadm.org/data.html).

The third source of data was a set of AC Maps, provided by the Data{Meet} Community Maps Project, made available under the Creative Commons Attribution 2.5 India. These were downloaded in shapefile format. In this dataset, ACs in the states of Jammu and Kashmir, Jharkhand, Assam, Manipur, Nagaland, and Arunachal Pradesh appear to be pre-delimitation boundaries—consequently, some AC names in these states are incorrect or missing. Additionally, the union territories of Andaman and Nicobar Islands, Chandigarh, Dadra and Nagar Haveli, Daman and Diu, and Lakshadweep have no ACs since they do not have a state assembly [24].

### 2.2. Methods

The distribution of census indicators on village amenities and demographics across ACs cannot be derived from these three disparate data sources on their own. Therefore, a key methodological contribution of our paper is merging these data sources into a single enriched dataset containing census amenities and demographics data with AC identifiers for each village. We performed this synthesis in two steps. First, we linked 2011 census village boundary and demographics data from ML Infomap with AC shapefiles by performing an ArcGIS “Spatial Join”, a spatial overlay technique that assigns each village point to the AC whose polygon it falls within. This spatial join manifested an additional column in the census data containing the village’s corresponding AC. Second, we merged the resulting 2011 census village boundary and demographics data with AC identifiers (containing 597,626 villages) with the village amenities dataset (containing 597,618 villages) using a sixteen-digit unique code constructed from state, district, sub-district, and village identifiers. This merge yielded a dataset with 597,121 villages, of which 5421 villages were excluded for missing education infrastructure data. Therefore, our final analytic sample contained 591,700 villages from 3773 ACs and all 36 states. We provide a map showing the number of villages within each AC across India to aid in interpreting our reported AC-level results (Figure S1).

### 2.3. Indicators of Education Infrastructure

We considered a total of 11 indicators of education infrastructure from the census village amenities data: Anganwadi centers; government pre-primary, primary, middle, secondary, and senior secondary schools; and private pre-primary, primary, middle, secondary, and senior secondary schools (Table 1). The presence of these facilities in a village was recorded in three mutually exclusive binary indicator variables representing various levels of deprivation in accessibility, purely based on distance to the facility: (1) at least one facility present within a village (“InV”), (2) facility not present in a village *and* the nearest facility is within 5 km (“OV ≤ 5 km”), and (3) facility not present in a village *and* the nearest facility is over 5 km away (“OV > 5 km”).

### 2.4. Statistical Analysis

We generated AC-level estimates by computing the proportion of villages within each AC that fell into the three categories of deprivation in accessibility to each type of education facility. For example, when considering government primary schools, we calculated the proportion of villages in each AC where (1) there was at least one government primary school in the village (InV%), (2) the nearest government primary school was outside the village and within 5 km (OV ≤ 5 km%), and (3) the nearest government primary school was outside the village and over 5 km away (OV > 5 km%). The 3773 ACs were ranked separately by proportion of InV, OV ≤ 5 km, and OV > 5 km for each of the 11 Census indicators for education infrastructure. To analyze the distribution of education infrastructure, first, we calculated summary statistics (interquartile range) across all ACs in India and by state. Then we visualized the distribution of education infrastructure using choropleth maps in order to identify regions with the greatest deprivation (i.e., highest quartile of OV> 5 km %). Lastly, we calculated a correlation matrix to quantify the linear relationship between the distribution of all education infrastructures. All analyses were performed using Python version 2.7.15 (Python Software Foundation) and R version 3.5.1 (The R Foundation for Statistical Computing, Vienna, Austria).

## 3. Results

In the following subsections, we present for each indicator (1) a summary of variation in access to education infrastructure across all ACs, (2) a summary of the states with the greatest AC variation in access to education infrastructure, and (3) a description of the areas of India with greatest deprivation of access to education infrastructure. We provide a state-wise ranking of ACs for access to all education infrastructures in the supplementary information (Supplementary File 1), as well as graphical representations InV%, OV ≤ 5 km%, and OV > 5 km% across ACs (Figure S2).

### 3.1. Anganwadi Centers

**Figure 1:**
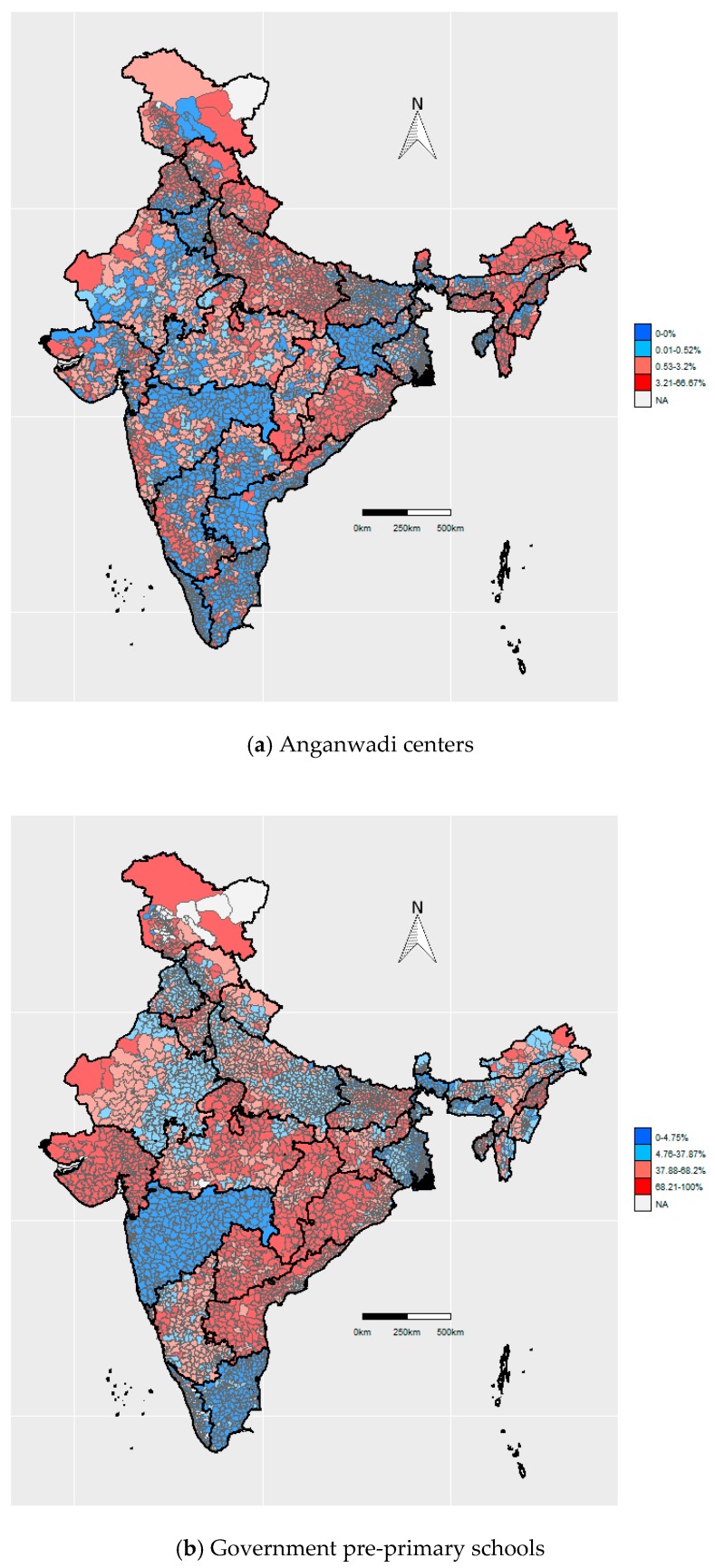

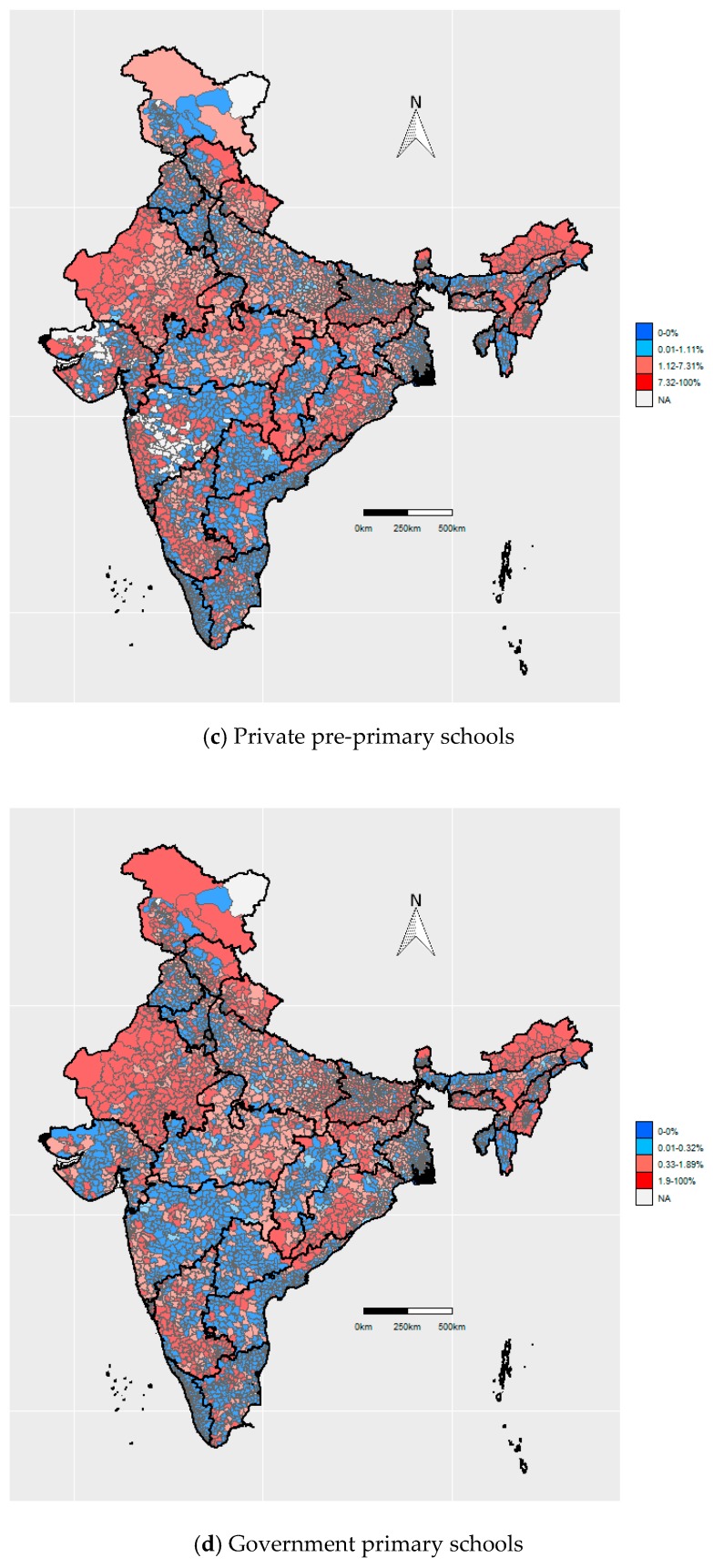

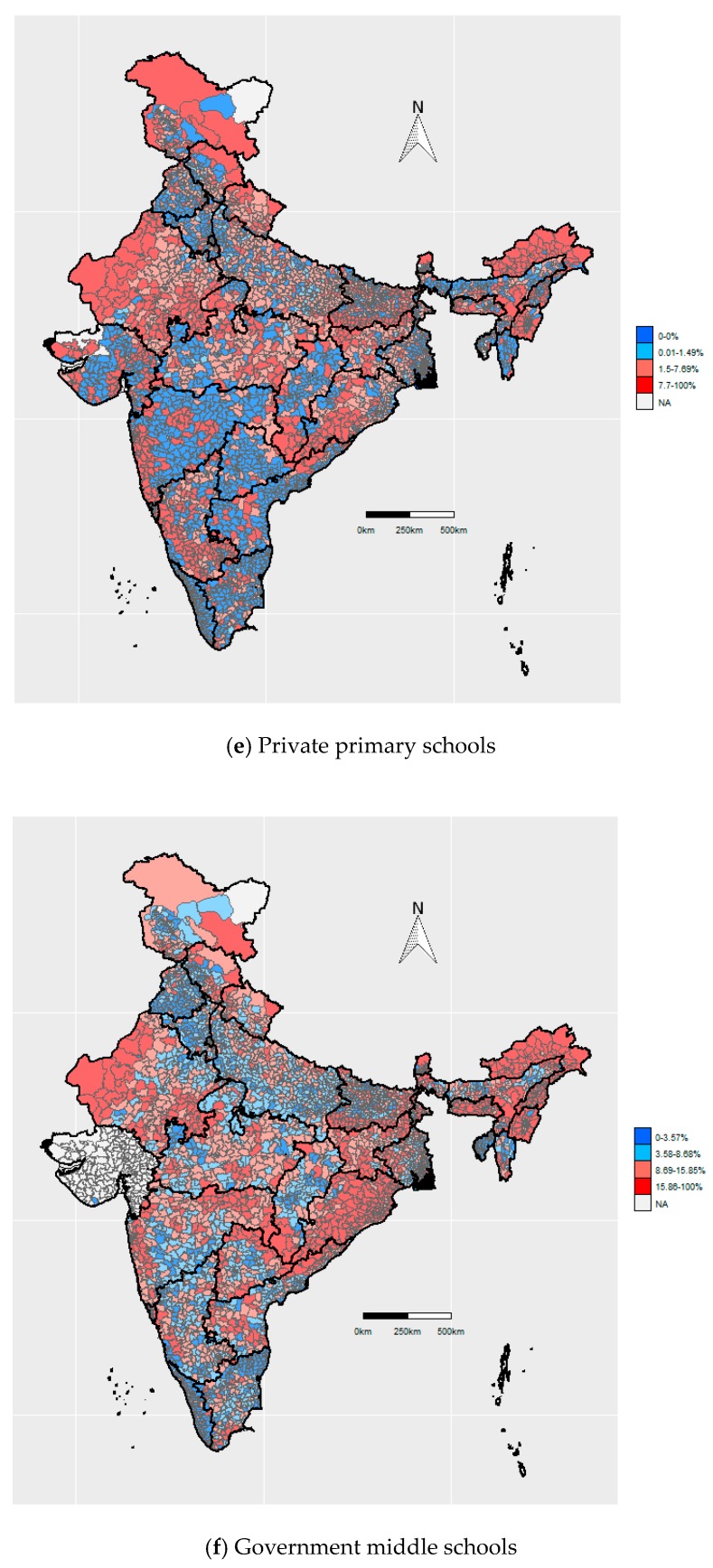

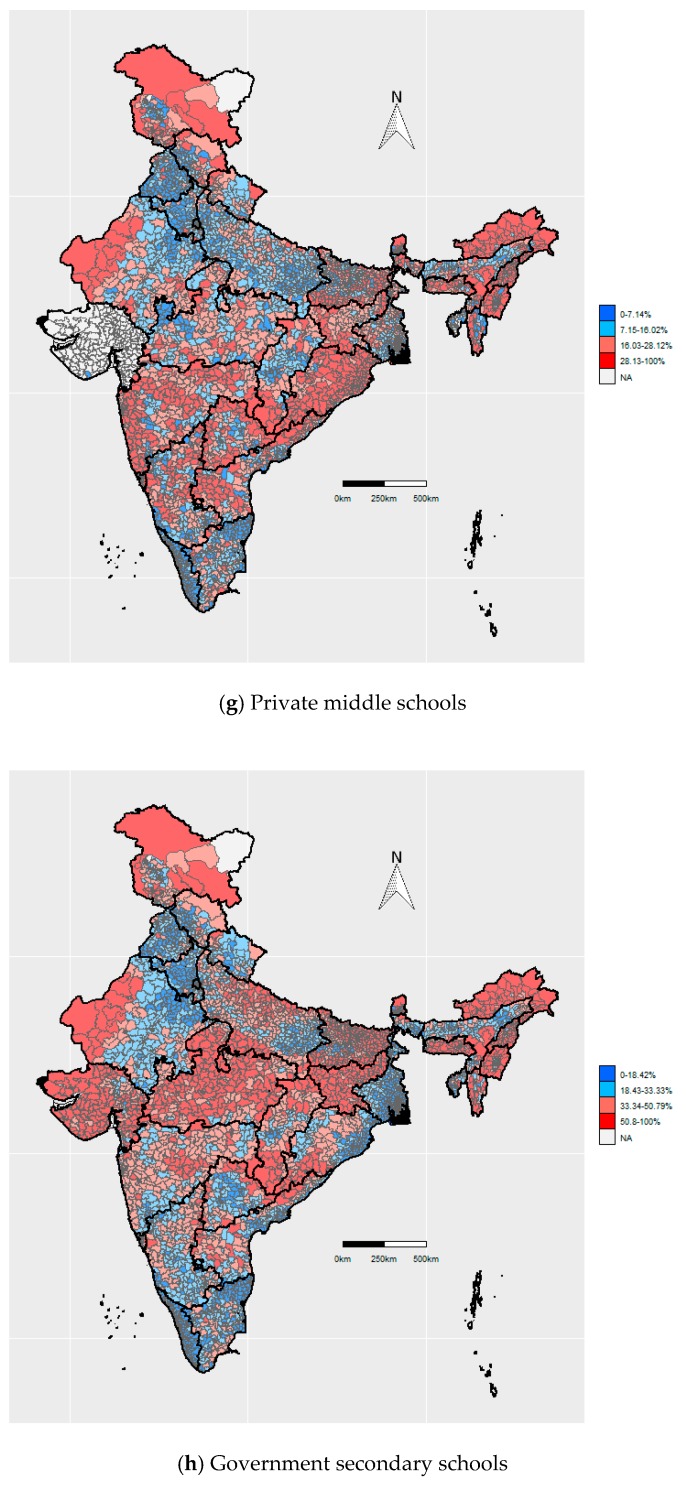

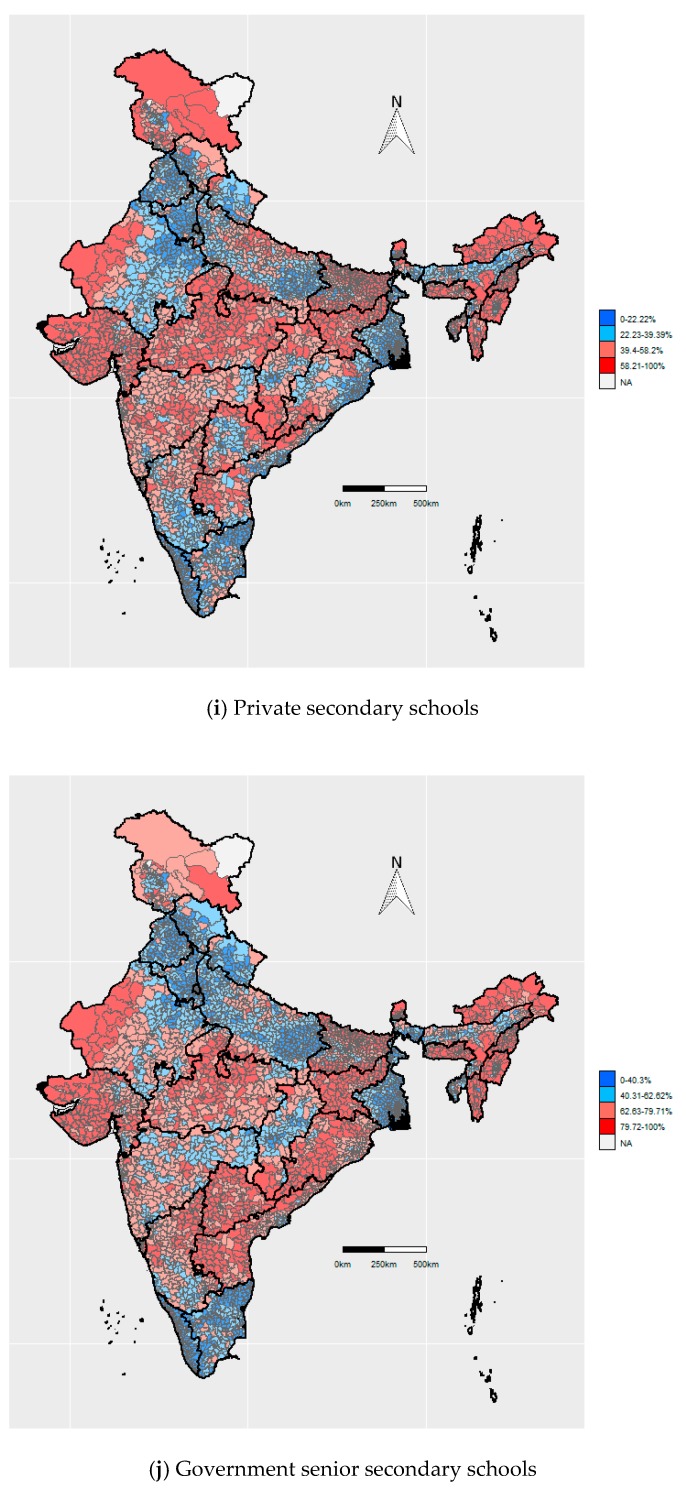

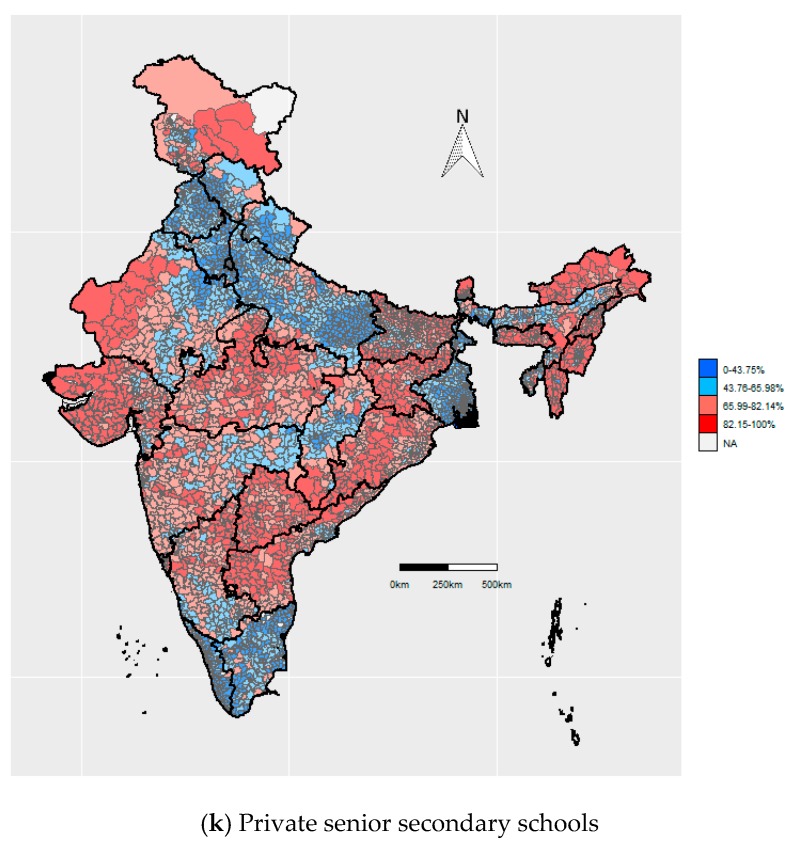
Maps showing OV > 5 km% (the percentage of villages where the nearest facility was outside the village and over 5km away) per AC. We show maps for Anganwadi centers (**a**), government pre-primary schools (**b**), private pre-primary schools (**c**), government primary schools (**d**), private primary schools (**e**), government middle schools (**f**), private middle schools (**g**), government secondary schools (**h**), private secondary schools (**i**), government senior secondary schools (**j**), and private senior secondary schools (**k**), Colors represent quartiles, ranging from blue (lowest deprivation) to red (highest deprivation).

Access to Anganwadi centers was consistently high across all ACs, with OV > 5 km% ranging from 0% at the 25th percentile to 3.2% at the 75th percentile (IQR: 3.2%) (Table 2). Similarly, InV% ranged from 85.5% at the 25th percentile to 99.4% at the 75th percentile. Despite low national variation in access to Anganwadi centers, certain states showed substantially higher variation in OV > 5 km%, namely Mizoram (IQR: 15.7%), Nagaland (IQR: 14.2%), and Arunachal Pradesh (IQR: 13.2%) (Table S1). The ACs in the bottom quartile (3.2%–66.7%) for OV > 5 km% were concentrated primarily in northern India (Jammu and Kashmir, Uttar Pradesh, Uttarakhand, Himachal Pradesh), and eastern India (Arunachal Pradesh, Meghalaya, Mizoram, Nagaland), as well as Odisha and Chhattisgarh (Figure 1a).

### 3.2. Pre-primary Schools

Access to government pre-primary schools varied substantially across ACs, with OV > 5 km% in an AC ranging from 4.8% (25th percentile) to 68.2% (75th percentile) (IQR: 63.4%) (Table 2). Bihar (IQR: 47.0%), Tripura (IQR: 46.7%), and Himachal Pradesh (IQR: 39.3%) showed high variation in OV > 5 km% across ACs (Table S1). The ACs in the bottom quartile (68.2%–100%) for OV > 5 km% were concentrated primarily in central India (Gujarat, Madhya Pradesh, Chhattisgarh, Odisha, Jharkhand), and southern India (Andhra Pradesh, Telangana) (Figure 1b).

Private pre-primary schools were consistently more accessible than their government counterparts. OV > 5 km% ranged from just 0% (25th percentile) to 7.3% (75th percentile) (IQR: 7.3%) (Table 2). Similarly, InV% ranged anywhere from 42.3% (25th percentile) to 93.4% (75th percentile). Certain states showed considerably greater AC variation in access to private pre-primary schools compared to the national-level AC variation. Gujarat (IQR: 50.0%), Maharashtra (IQR: 40.0%), and Arunachal Pradesh (IQR: 31.5%) showed the highest variation in OV > 5 km% (Table S1). The ACs in the bottom quartile (7.3%–100%) for OV > 5 km% were concentrated primarily in Arunachal Pradesh, Rajasthan, and central India (Figure 1c).

### 3.3. Primary Schools

AC-level access to government primary schools was consistently high, with OV > 5 km% ranging from 0% (25th percentile) to 1.9% (75th percentile) (IQR: 1.9%) (Table 2). Similarly, InV% ranged from 85.0% (25th percentile) to 99.0% (75th percentile). Despite low variation at the national level, certain states showed higher variation in OV > 5 km%, such as Arunachal Pradesh (IQR: 25.5%), Nagaland (IQR: 8.3%), and Meghalaya (IQR: 6.2%) (Table S1). The ACs in the bottom quartile (1.9%–100%) for OV > 5 km% were concentrated primarily in northern India (Jammu and Kashmir, Himachal Pradesh, Uttarakhand), central India (Rajasthan, Odisha), and eastern India (Arunachal Pradesh, Meghalaya) (Figure 1d).

The variation in OV > 5 km% for private primary schools was also low, ranging from 0% (25th percentile) to 7.7% (75th percentile) across all ACs (IQR: 7.7%) (Table 2). Certain states had notably greater variation than the national IQR, such as Arunachal Pradesh (IQR: 37.0%), Nagaland (IQR: 22.6%), and Bihar (IQR: 19.7%) (Table S1). The ACs in the bottom quartile (7.7%–100%) for OV > 5 km% were concentrated primarily in northern India (Jammu and Kashmir, Himachal Pradesh, Uttarakhand), central India (Rajasthan), and eastern India (Arunachal Pradesh, Meghalaya) (Figure 1e).

### 3.4. Middle Schools

We excluded Gujarat from our discussion of middle schools due to excessive missing data. OV > 5 km% for government middle schools varied from 3.6% (25th percentile) to 15.9% (75th percentile) across all ACs (IQR: 12.3%) (Table 2). Manipur (IQR: 48.9%), Arunachal Pradesh (IQR: 28.4%) and Nagaland (IQR: 25%) all had greater within-state variation in OV > 5 km% than reflected in the national IQR (Table S1). The ACs in the bottom quartile (7.7%–100%) for OV > 5 km% were concentrated primarily in central and eastern India (Rajasthan, Odisha, Jharkhand, Arunachal Pradesh, Meghalaya, Nagaland) (Figure 1f).

OV > 5 km% for private middle schools was anywhere from 7.1% (25th percentile) to 28.1% (75th percentile) (IQR: 21.0%) (Table 2). Certain states showed greater within-state variation in access to private middle schools compared to the national IQR, such as Manipur (IQR: 50.0%), Mizoram (IQR: 41.8%), and Nagaland (29.9%) (Table S1). The ACs in the bottom quartile (28.13%–100%) for OV > 5 km% were concentrated primarily in central (Odisha, Jharkhand), southern (Andhra Pradesh, Maharashtra, Karnataka), and eastern India (Arunachal Pradesh, Meghalaya, Nagaland) (Figure 1g).

### 3.5. Secondary Schools

Access to government secondary schools varied substantially across ACs, with OV > 5 km% ranging from 18.4% (25th percentile) to 50.8% (75th percentile) (IQR: 32.4%) (Table 2). Manipur (IQR: 66.3%), Bihar (IQR: 36.9%), and Mizoram (IQR: 33.0%) showed the highest variation in OV > 5 km% (Table S1). The ACs in the bottom quartile (50.8%–100%) for OV > 5 km% were concentrated primarily in central India (Gujarat, Madhya Pradesh, Jharkhand, Bihar, Chhattisgarh, Uttar Pradesh) and eastern India (Arunachal Pradesh, Nagaland, Manipur) (Figure 1h).

OV > 5 km% for private secondary schools varied from 22.2% (25th percentile) to 58.2% (75th percentile) (IQR: 36.0%) (Table 2). Certain states, including Manipur (IQR: 65.3%), Bihar (IQR: 40.6%), and Tripura (IQR: 40.4%) showed higher within-state variation in access to private secondary schools compared to the national IQR (Table S1). The ACs in the bottom quartile (58.2%–100%) for OV > 5 km% were concentrated primarily in central India (Gujarat, Madhya Pradesh, Jharkhand, Bihar, Chhattisgarh), eastern India (Arunachal Pradesh, Nagaland, Manipur), and southern India (Maharashtra, Andhra Pradesh) (Figure 1i).

### 3.6. Senior Secondary Schools

Access to government senior secondary schools was consistently poor, with an OV > 5 km% of 40.3% at the 25th percentile and 79.7% at the 75th percentile (IQR: 39.4%) (Table 2). Similarly, InV% varied from just 2.4% (25th percentile) to 11.5% (75th percentile). The states with the highest variation in OV > 5 km% were Manipur (IQR: 51.0%), Sikkim (IQR: 37.5%), and Tamil Nadu (IQR: 35.4%) (Table S1). The ACs in the bottom quartile (79.7%–100%) for OV > 5 km% were concentrated primarily in central India (Gujarat, Madhya Pradesh, Jharkhand, Bihar, Odisha), eastern India (Arunachal Pradesh, Nagaland, Manipur, Mizoram), and southern India (Telangana, Andhra Pradesh, Karnataka) (Figure 1j).

OV > 5 km% for private senior secondary schools varied from 43.8% (25th percentile) to 82.1% (75th percentile) (IQR: 38.4%) (Table 2). InV% was extremely low, varying from 0% (25th percentile) to 5.5% (75th percentile). Manipur (IQR: 50.72%), Kerala (IQR: 42.9%), and Sikkim (IQR: 36.2%) showed the greatest within-state variation for OV > 5 km% (Table S1). The ACs in the bottom quartile (82.1%–100%) for proportion of OV > 5 km government senior secondary schools were concentrated primarily in central India (Gujarat, Madhya Pradesh, Jharkhand, Bihar, Odisha), eastern India (Arunachal Pradesh, Nagaland, Manipur, Mizoram), and southern India (Telangana, Andhra Pradesh, Karnataka) (Figure 1k).

### 3.7. Relationship across all Education Infrastructures

**Figure 2:**
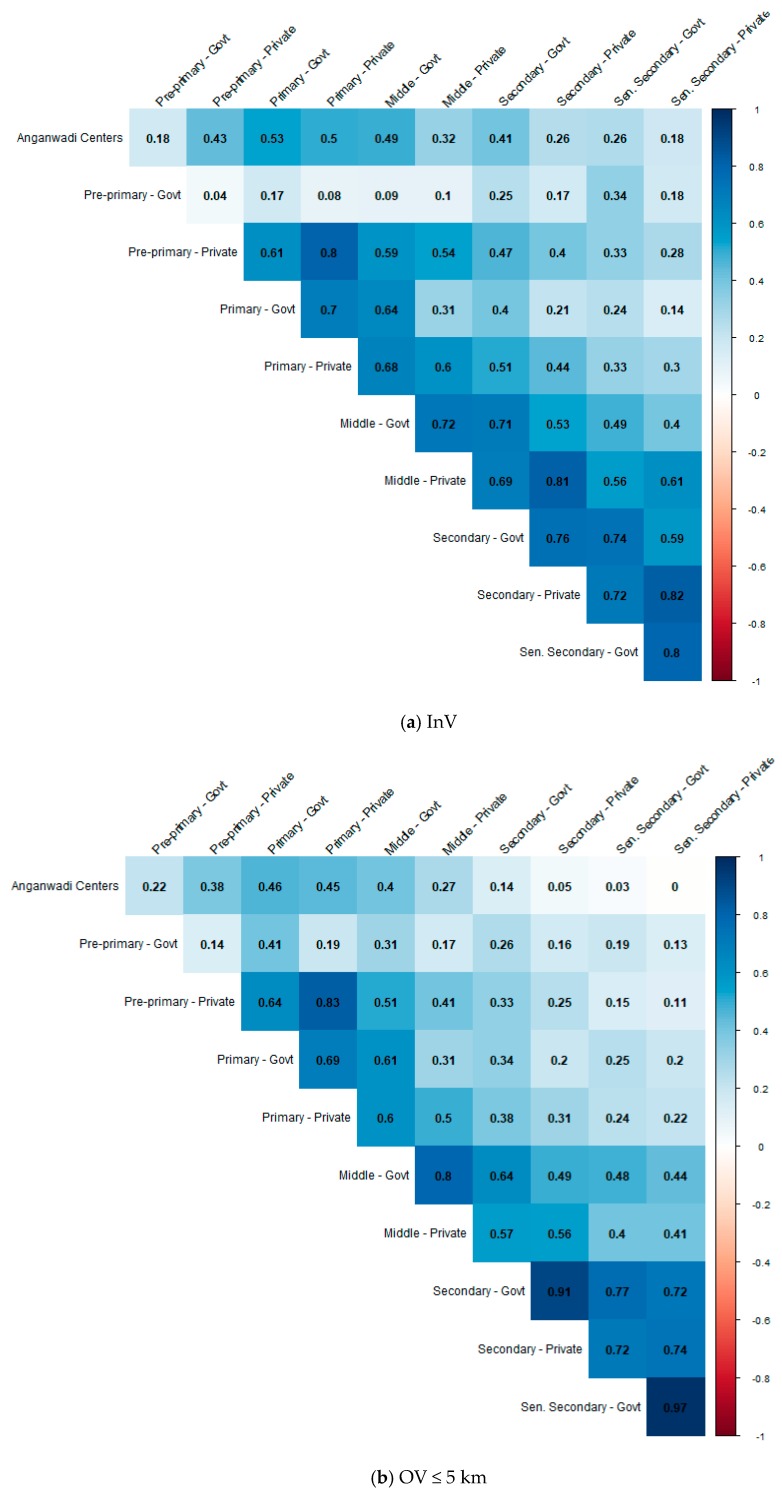

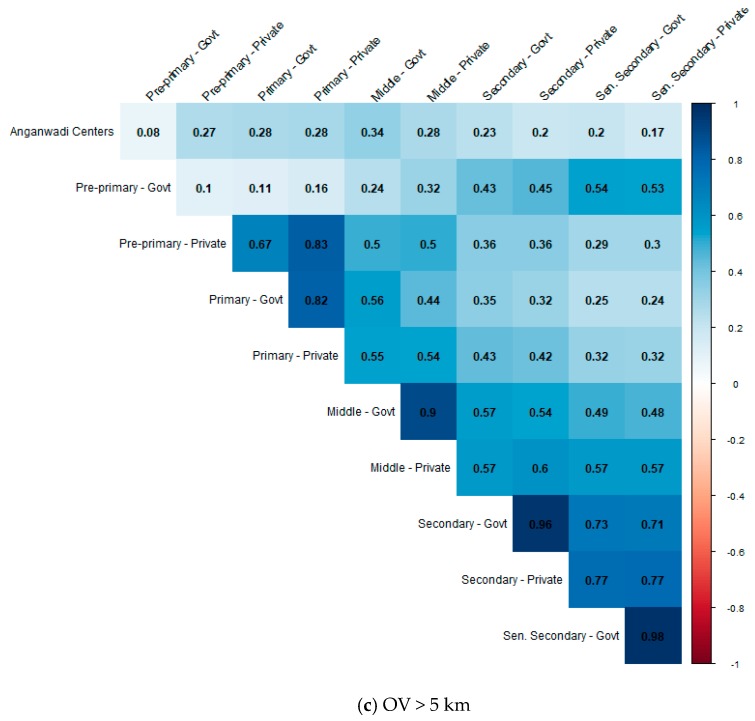
Pearson correlations between the distributions of the 11 education infrastructures at the AC level. Reported values represent correlations for the proportion of InV (**a**), OV ≤ 5 km (**b**), and OV > 5 km (**c**) in an AC.

In general, the proportion of OV > 5 km for government education infrastructures was strongly correlated with that of their private counterparts (r = 0.82 for primary school, r = 0.9 for middle school, r = 0.96 for secondary school, r = 0.98 for senior secondary school) (Figure 2). The exception was the distribution of government and private pre-primary schools, which had no correlation (r = 0.1). In general, correlations were strongest for adjacent levels of education and weaker for other levels of education (r = 0.56 for government primary and middle; r = 0.57 for government middle and secondary; r = 0.73 for government secondary and senior secondary). For example, the distribution of government primary schools was correlated with the distribution of government middle schools (r = 0.51), and progressively less correlated with government secondary schools (r = 0.35) and government senior secondary schools (r = 0.25). The exception was the distribution of government pre-primary schools, which was correlated with that of government senior secondary schools (r = 0.54), and progressively less correlated with that of lower level education infrastructure.

## 4. Discussion

Four salient findings emerged from our analysis that are relevant for AC-level education policy in rural India. First, we demonstrated that it is possible to derive AC-level estimates from village-level data using GIS overlay analysis and provided, for the first time, measures of AC-level access to education infrastructure. Second, we found high variability in access to higher education infrastructure (middle, secondary, and senior secondary schools) and low variability in access to lower education variables (Anganwadi centers, pre-primary schools, and primary schools). Third, we found substantial variability in access to various education infrastructures across states, with the highest variability in the states of Manipur, Arunachal Pradesh, and Bihar. Fourth, in general we found higher variation in access to private education infrastructure compared to government education infrastructure.

Aggregating data from lower-level to higher-level units can lead to loss of information, masking of lower-level heterogeneity, and problems of interpretation as seen in the Modifiable Areal Unit Problem and the Ecological Fallacy. The Modifiable Areal Unit Problem suggests that data estimates aggregated to higher-level units are highly dependent on the specific borders that segment the underlying units. In the context of this study, AC-level estimates of access to education infrastructure are indeed dependent on AC borders and the specific villages that fall within them. However, our units are not “modifiable” since we use official AC borders to group villages, as opposed to defining our own custom borders for higher-level unit analysis. In other words, since ACs have a priori sociopolitical relevance, we take ACs as our unit of analysis and thus avoid bias that would arise from defining our own modifiable areal units. The Ecological Fallacy suggests that the nature of individuals can be wrongly deduced by observing trends within a larger group. We mitigate potential misinterpretation of this kind by reporting the proportion of villages within an AC that fall into various distance groups, rather than calculating AC-level means that would mask variance within ACs. Furthermore, when reporting state-level results, we exclusively report measures of variability like IQR instead of mean and median, which may mask state-level heterogeneity. In doing so, we retain the underlying village data and mitigate the loss of information upon aggregation.

Our analysis faces important data limitations. First, since we used data from the 2011 India Census, our estimates may not reflect current access to education infrastructure. We recommend a similar analysis to be done when the next Census becomes available to assess improvements in access to education infrastructure across all ACs, and by states. Second, the composition of the Census Village Amenities files restricted our analysis to rural villages, and hence we cannot extend our findings to urban areas. Therefore, we caution the readers when interpreting our AC estimates, which are only indicative of the rural areas within each AC. Third, the scope of our analysis is restricted to the descriptive distribution of education infrastructure at the AC level. Since explaining the regional and other variation in our findings would require deeper investigation of the local context, we provide the AC-level estimates as a springboard for future studies. With regards to our geospatial methodology for calculating AC estimates, the effectiveness of spatial overlay analysis techniques such as spatial join are dependent on the mapping accuracy of the features being compared. The village and AC maps provided by ML Infomap and Data{Meet} Community Maps Project were visually compared to high resolution satellite imagery. However, due to the size of India not all areas could be assessed for accuracy. Lastly, we defined levels of accessibility to educational facilities in terms of geographic distance, and do not consider other social and economic barriers to school attendance. Even where access is guaranteed, other pressing challenges like teacher absenteeism, high dropout rates, and poor-quality instruction can hinder a child’s education [23]. In the absence of data on quality of education and socioeconomic factors at the AC level, we provide a crude assessment of accessibility to education infrastructure as an important step toward positive educational outcomes. Our methodology can also be extended to generate AC-level sociodemographic estimates, supporting future analyses of risk factors for school achievement.

The substantial between-AC variability in several education infrastructure-related indicators, both nationwide and within states, may be explained by several factors. For instance, the AC variability on the national level may be explained by historical economic disparities in different regions. More economically advanced states tend to be more socially cohesive, spend more on social and developmental programs, and have better governance and laws surrounding public expenditures [25]. Within-state disparities can perhaps be explained by inconsistent spending patterns of MLAs within states. For instance, in New Delhi in 2014, at least six MLAs were reported of having crores of unused MLAADS funds, while over a dozen other MLAs had spent all of theirs [26]. Similarly, in Telangana in 2016, only 30–40% of funds allocated for AC interventions were being used [27]. Our work provides the baseline data needed for follow-up studies to investigate why specific ACs are doing poorly in terms of accessibility to education infrastructure. This intrastate variance also calls into question how states oversee the usage of funds across ACs. Currently, state legislatures have PACs to make policy recommendations and streamline expenditures of state politicians [28]. However, without a way to link fact-based insights from Census and other population surveys to ACs, PACs cannot make data-driven policy recommendations to MLAs. This highlights the practical need for the methodologies provided in this paper.

It is important to note that while both MLAs and MPs have important legislative roles, their administrative scopes differ. For certain policy issues like education, which straddle both MLA and MP domains, national level policy—such as the ICDS program—may be determined by MPs, while local implementation—such as the construction of ICDS centers—may be led by MLAs. Since we focused on the distribution of education infrastructure across rural India, our study centered around ACs and MLAs, who shoulder greater accountability than MPs for the condition of local education infrastructure.

Our findings show higher variability in access to higher level education infrastructure and lower variability for lower level education infrastructure. This pattern may reflect the impact of national education policies. For example, uniformly high access to primary schools across ACs may reflect the effects of targeted initiatives like the Right to Education Bill, which provides universal education access until age 14 [29]. Similarly, the 2001 Sarva Shiksha Abhiyan campaign expanded the number of primary schools in the country from 845,007 in 2000–2001 to 1,042,251 in 2004–2005 [30]. More recently, programs to increase access to secondary schools have sprung up, such as the Rastriya Madhyamic Shiksha Abhiyan. Launched in 2009, the program aimed to achieve universal access to secondary education in 2017 and improve secondary school access by ensuring there was a secondary school “within a reasonable distance of any habitation” [31]. Though this program is coordinated by the Ministry of Human Resource Development, individual states have a large role in its implementation logistics and financing, which perhaps explains disparities at the state and AC level [31]. A prior analysis of state spending showed that while most states spend more on elementary education than secondary, Kerala, Maharashtra, West Bengal, Tamil Nadu, and Punjab spent as much, if not more, on secondary education [29]. We found that Kerala, West Bengal, Tamil Nadu, and Punjab were 4 of the 5 states with the lowest variation for secondary school access, suggesting that higher spending on secondary education may be associated with lower variation in access across ACs.

On average, private schools were less accessible than government schools. There was higher variability across ACs in access to private education infrastructure than in access to government infrastructure. The exception to this pattern was pre-primary education, where access to government pre-primary schools was lower on average and showed substantially greater variation than access to private pre-primary schools. This is perhaps due to the fact that children have several options for pre-primary education, including Anganwadi centers, government pre-primary schools, and private pre-primary schools. Indeed, only 6% of pre-primary-age children who are enrolled in school attend government pre-primary schools, compared to 78% who attend Anganwadi centers and 15% who attend private pre-primary schools [11]. Private schools have seen rising enrollment in the past few decades, especially in rural areas [17]. All India private school enrollment increased from 18.7% in 2006 to 30.9% in 2018 (ASER, 2018). One study on primary education found that in rural areas, private schools are utilized as an improved alternative to failing public schools. Private schools were found to pay one-fifth the salary of public schools on average, allowing them to hire more teachers and thus have lower student-teacher ratios [17]. If current trends continue, private schools could be an important resource to a substantial proportion of Indian children.

## 5. Conclusions

ACs in India are important geopolitical units for state-level legislature but —largely due to a lack of data—remain unexplored in empirical assessments of population health and development. Our analysis of the distribution in accessibility to education infrastructure across ACs revealed high variability in access to higher education infrastructures (middle, secondary, and secondary schools), low variability in access to lower education infrastructures (Anganwadi centers, private pre-primary schools, and primary schools), substantial variability in access to various education infrastructures across states, and higher variation in access to private education infrastructures compared to government education infrastructure. These findings are highly relevant for diverse stakeholders, including MLAs, policymakers, PACs, researchers, and constituents. Just as the “Transformation of Aspirational Districts” program ranked districts to guide resource allocation and foster collaboration and competition among districts [2], our AC ranking can introduce a healthy level of collaboration and competition to the AC-level education space, providing tools and evidence for MLAs and political leaders to conduct more informed and targeted resource allocation. Our findings also call for future studies to investigate the ACs and states with the highest variation in access to education infrastructures. Deeper investigation of the local context can lead to more specific and tailored policy recommendations. Importantly, our methodology can be applied to diverse datasets to calculate AC-level estimates on a wide array of indicators, including access to other amenities such as health centers or water infrastructure, or measures of human capital like literacy rate, poverty, and employment rate. Furthermore, the idea behind our methodology—combining existing datasets and geographic shapefiles for policy use—can be extended to any geographic region or context where data-driven policy is lacking. Our work provides the foundation for data-driven policy with the power to hold elected representatives accountable for the wellbeing of their constituents.

## Figures and Tables

**Table 1:** Definitions provided by the 2011 Indian Census for the education infrastructures that we considered in our analysis.

Term	Definition Provided by Indian Census 2011
Anganwadi center	Our definition of Anganwadi center includes the two definitions provided by the census below: (1) Nutrition Centre: Integrated Child Development Services (ICDS): The Integrated Child Development Services (ICDS) Scheme set up by the Government of India with the objective of providing following package of services to the children under 6 years and pregnant and lactating mothers in villages such as: immunization, health check-up, referral services, pre-school nonformal education and nutrition & health education. (2) Anganwadi Centre: Each centre under the ICDS scheme is run by an Anganwadi Worker. One Anganwadi worker is appointed for specified population of the village. They are basically local women. They are assisted by Anganwadi helper. They provide pre-school non-formal education at the centre and provide food to the children.
Pre-Primary school	Nowadays, the children are sent to schools at a very early age. Lot of pre-primary schools, private schools in particular, have been opened in villages and towns. These may or may not be recognized by the competent authorities. Even many secondary schools have classes starting from the pre-primary level. Pre-primary classes include nursery, K.G., pre-basic, playschool, etc.
Primary school	Schools providing education from Standard 1 and upward up to and inclusive of Standard V are classified as primary schools.
Middle school	Schools providing education from Standard VI and upward up to and inclusive of Standard VIII are classified as middle schools. A school with Class 1 to VIII is treated as two units, i.e., one primary school and one middle school.
Secondary school	Schools providing education from Standard IX and upwards up to and inclusive of Standard X are classified as secondary schools. A composite school with 1 to X standard is treated as three separate units and counted separately under the categories of primary school, middle school and secondary school.
Senior Secondary school	Schools and colleges that provide education for Standards XI and XII and first and second year of the pre-university course fall under this category. There are Senior secondary schools with Standard I and upwards up to Standard XII.

**Table 2:** The interquartile range for percentage of villages where there is at least 1 education infrastructure in the village (InV%), the nearest education infrastructure is outside the village and within 5 km (OV ≤ 5 km%), and the nearest education infrastructure is outside the village and over 5 km away (OV > 5 km%).

	InV (%)	OV ≤ 5 km (%)	OV > 5 km (%)
Anganwadi Center	85.49–99.38	0–9.68	0–3.2
Pre-primary—Govt.	0–70	6.02–48.73	4.75–68.2
Primary—Govt.	85.03–99.01	0–11.78	0–1.89
Middle—Govt.	39.17–72.75	17.14–44.79	3.57–15.85
Secondary—Govt.	9.32–33.33	24.2–54.13	18.42–50.79
Senior Sec.—Govt.	2.36–11.54	13.56–44	40.3–79.71
Pre-primary—Private	42.31–93.6	2.91–44.44	0–7.31
Primary—Private	43.48–92.86	3.57–45	0–7.69
Middle—Private	7.4–38.73	36.58–71.22	7.14–28.12
Secondary—Private	1.96–14.62	29.89–61.71	22.22–58.2
Senior Sec.—Private	0–5.45	14.29–46.41	43.75–82.14

## Supplementary Materials

The following are available online at https://www.mdpi.com/1660-4601/17/1/296/s1, Supplementary File 1: For each AC, the proportion of InV, OV ≤ 5 km, and OV > 5 km, as well as the within-state ranking of the AC by proportion of InV, OV ≤ 5 km, and OV > 5 km. Figure S1: Map showing the number of villages within each AC across India. Colors represent number of villages, ranging from blue (lowest number) to red (highest number). Figure S2: Stacked bar plots showing the village-level distribution of education infrastructures across 3719 rural ACs of India. 1 = At least one facility in village (InV), 2 = 0 facilities in village and nearest facility <=5 km away (OV <= 5 km), 3 = 0 facilities in village and nearest facility >5 km away (OV > 5 km). We report show the village-level distribution for Anganwadi centers (a), government pre-primary schools (b), private pre-primary schools (c), government primary schools (d), private primary schools (e), government middle schools (f), private middle schools (g), government secondary schools (h), private secondary schools (i), government senior secondary schools (j), and private senior secondary schools (k). Table S1: For each state, the interquartile range in the proportion of InV (at least one facility is present in the village) (a), OV ≤ 5 km (facility not present in a village and the nearest facility within 5 km) (b), and OV > 5 km (facility not present in a village and the nearest facility over 5 km away) (c) across all ACs for 11 different education infrastructures.

## References

[1] AbouZahr C., De Savigny D., Mikkelsen L., Setel P.W., Lozano R., Nichols E., Notzon F., Lopez A.D. (2015). Civil registration and vital statistics: Progress in the data revolution for counting and accountability. Lancet.

[2] NITI Aayog (2018). Transformation of Aspirational Districts..

[3] Kim R., Swaminathan A., Kumar R., Xu Y., Blossom J.C., Venkataramanan R., Kumar A., Joe W., Subramanian S.V. (2019). Estimating the burden of child malnutrition across parliamentary constituencies in India: A methodological comparison. Ssm-Popul. Health.

[4] Swaminathan A., Kim R., Xu Y., Blossom J.C., Venkatraman R., Kumar A., Subramanian S.V. (2019). Burden of Child Malnutrition in India: A view from parliamentary constituencies. Econ. Political Wkly..

[5] Maheshwari S. (1976). Constituency Linkage of National Legislators in India. Legis. Stud. Q..

[6] Rural Development and Panchayat Raj Department (2014). Member of Legislative Assembly Constituency Development Scheme (MLACDS).

[7] Singh V., Gehlot B., Start D., Johnson C. (2003). Out of Reach: Local Politics and the Distribution of Development Funds in Madhya Pradesh.

[8] Kingdon G.G. (2007). The progress of school education in India. Oxf. Rev. Econ. Policy.

[9] Das D., Pathak M. (2012). The growing rural-urban disparity in India: Some issues. Int. J. Adv. Res. Technol..

[10] Fan S., Hazell P., Thorat S. (2000). Government spending, growth and poverty in rural India. Am. J. Agric. Econ..

[11] ASER (2018). Annual Status of Education Report (Rural).

[12] UNESCO Data for the Sustainable Development Goals 2019: India. http://uis.unesco.org/en/country/in?theme=education-and-literacy.

[13] Pattnaik J. (1996). Early childhood education in India: History, trends, issues, and achievements. Early Child. Educ. J..

[14] Mittal N., Meenakshi J. (2015). Utilization of ICDS Services and Their Impact on Child Health Outcomes: Evidence from three East Indian states.

[15] Kingdon G., Unni J. (2001). Education and Women’s Labor Market Outcomes in India. Educ. Econ..

[16] Rose P.M., Dyer C. (2008). Chronic Poverty and Education: A Review of Literature. Chronic Poverty Res. Centre.

[17] Muralidharan K., Kremer M. (2006). Public and Private Schools in Rural India.

[18] Nava F. (2009). Factors in School Leaving: Variations across gender groups, school levels and locations. Educ. Q..

[19] Gould W.T. (1978). Guidelines for School Location Planning.

[20] Paxson C., Schady N.R. (2002). The allocation and impact of social funds: Spending on school infrastructure in Peru. World Bank Econ. Rev..

[21] Duflo E. (2001). Schooling and labor market consequences of school construction in Indonesia: Evidence from an unusual policy experiment. Am. Econ. Rev..

[22] Birdsall N., Levine R., Ibrahim A. (2005). Towards Universal Primary Education: Investments, incentives, and institutions. Eur. J. Educ..

[23] Kingdon G., Banerji R. (2009). Addressing School Quality: Some Policy Pointers from Rural North India.

[24] ML Infomap (2017). Process of Creating Countrywide Village Boundary 7 Settlement Maps of India. https://www.mlinfomap.com/.

[25] Kurian N. (2007). Widening economic & social disparities: Implications for India. Indian J. Med. Res..

[26] Kant V. (2014). Crores of MLALAD Fund lie unused. The Hindu.

[27] Mahesh K. (2016). MLAs stingy in spending funds. Times of India.

[28] Chopra V.K. (1994). Legislators in India: A Comparison of MLAs in Five States. Doctoral dissertation.

[29] Lewin K.M. (2011). Expanding access to secondary education: Can India catch up?. Int. J. Educ. Dev..

[30] Govinda R. (2008). Education for all in India: Assessing progress towards Dakar Goals. Prospects.

[31] Ministry of Human Resource Development (2016). Rashtriya Madhyamik Shiksha Abhiyan (RMSA).

