# Supplementary material for: The State of School Infrastructure in the Assembly Constituencies of Rural India: Analysis of 11 Census Indicators from Pre-primary to Higher Education
Source: Int J Environ Res Public Health. 2020 Jan 1;17(1):296. doi: 10.3390/ijerph17010296 (PMC6981364; doi:10.3390/ijerph17010296)
Supplement: Supplementary file 1 [file ijerph-17-00296-s001.zip › IJERPH-644555-Supplementary File 2 1-2-20.docx]

**Supplementary Materials**

**The State of School Infrastructure in the Assembly Constituencies of Rural India: Analysis of 11 Census Indicators from Pre-primary to Higher Education**

**Figure S1:** Map showing the number of villages within each AC across India. Colors represent number of villages, ranging from blue (lowest number) to red (highest number).

**Figure S2**: Stacked bar plots showing the village-level distribution of education infrastructure across 3719 rural ACs of India. 1 = At least one facility in village (InV), 2 = 0 facilities in village and nearest facility <=5km away (OV<=5km), 3 = 0 facilities in village and nearest facility >5km away (OV>5km). We report show the village-level distribution for Anganwadi centers (a), government pre-primary schools (b), private pre-primary schools (c), government primary schools (d), private primary schools (e), government middle schools (f), private middle schools (g), government secondary schools (h), private secondary schools (i), government senior secondary schools (j), and private senior secondary schools (k).

**Table S1**: For each state, the interquartile range in the proportion of InV education facilities (at least one facility is present in the village) (a), OV≤5km education facilities (facility not present in a village and the nearest facility within 5km) (b), and OV>5km education facilities (facility not present in a village and the nearest facility over 5km away) (c) across all ACs for 11 different educational infrastructures.

**Figure S1:** Map showing the number of villages within each AC across India. Colors represent number of villages, ranging from blue (lowest number) to red (highest number).

**
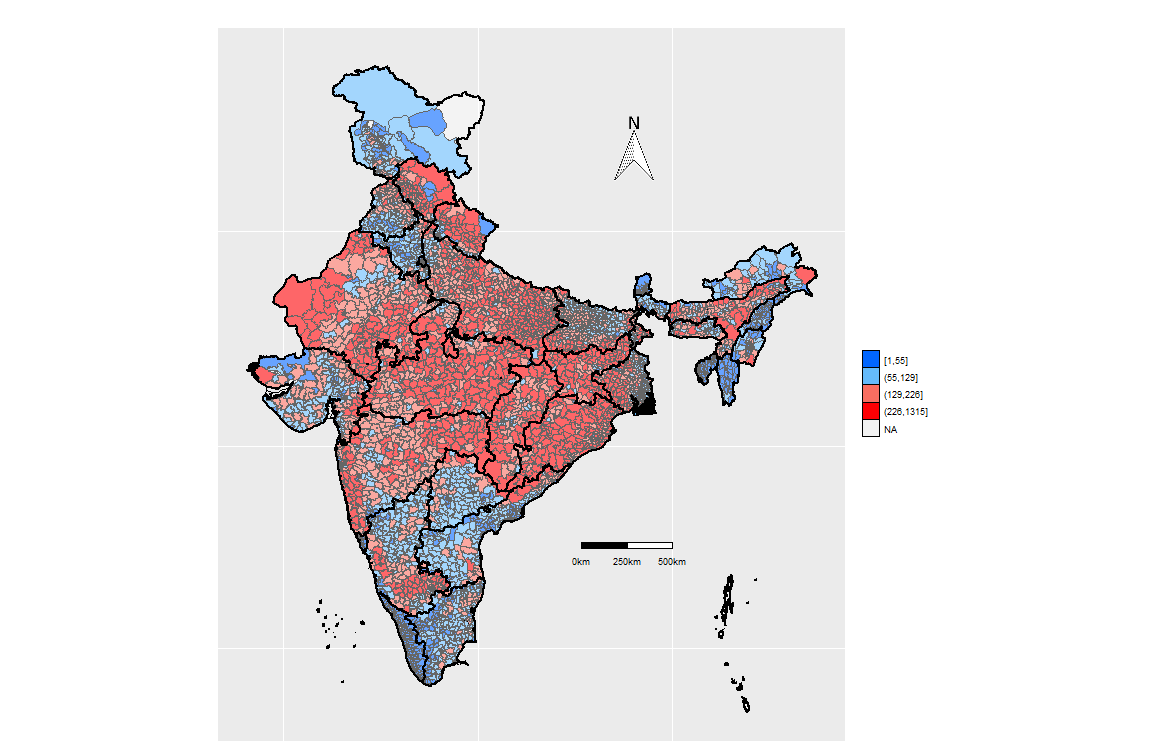
**

**Figure S2:** Stacked bar plots showing the village-level distribution of education infrastructure across 3719 rural ACs of India. 1 = At least one facility in village (InV), 2 = 0 facilities in village and nearest facility <=5km away (OV<=5km), 3 = 0 facilities in village and nearest facility >5km away (OV>5km). We report show the village-level distribution for Anganwadi centers (a), government pre-primary schools (b), private pre-primary schools (c), government primary schools (d), private primary schools (e), government middle schools (f), private middle schools (g), government secondary schools (h), private secondary schools (i), government senior secondary schools (j), and private senior secondary schools (k).

(a) anganwadi centers
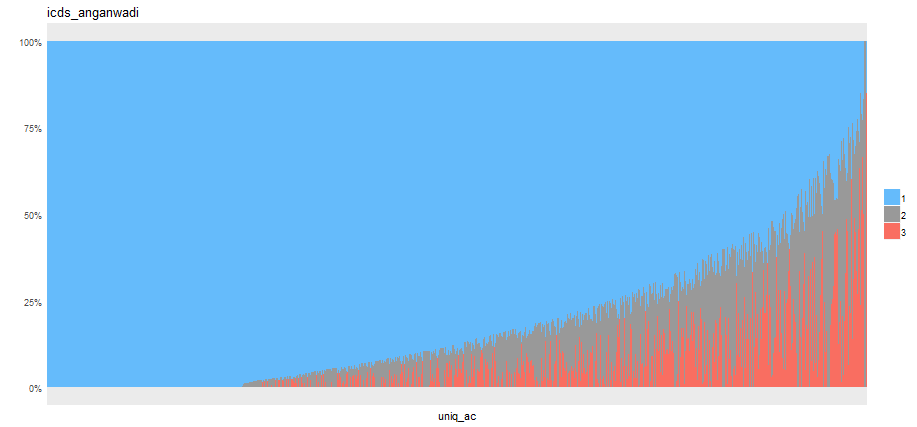


(b) government pre-primary schools


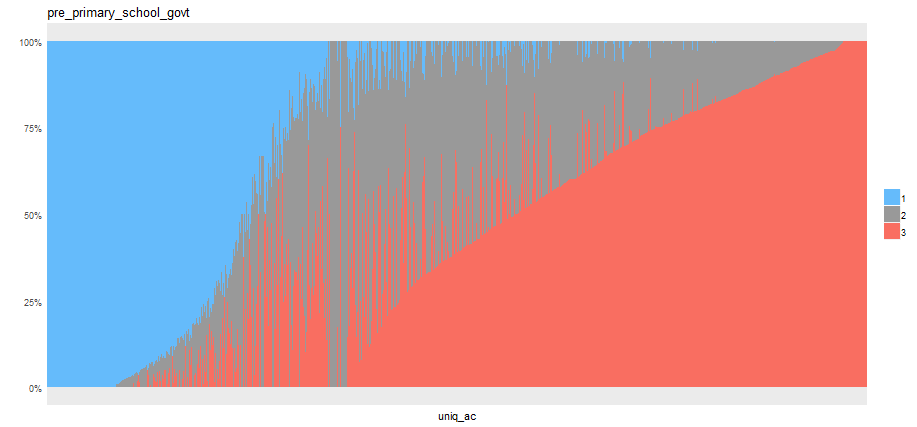


(c) private pre-primary schools


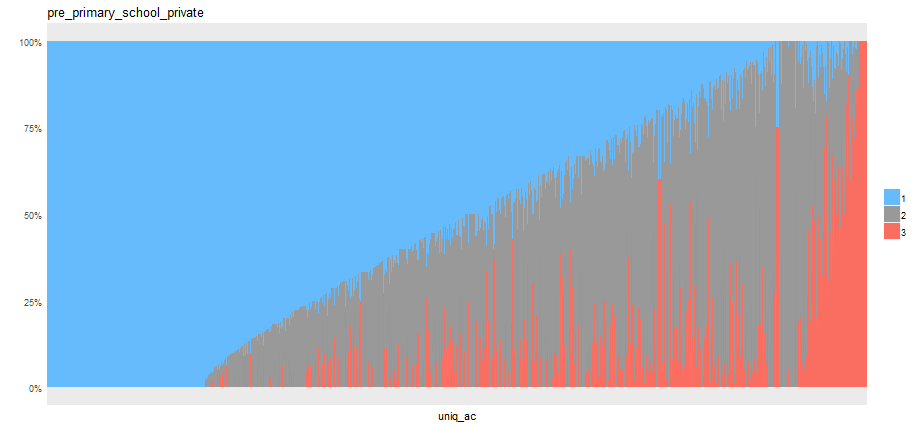


(d) government primary schools


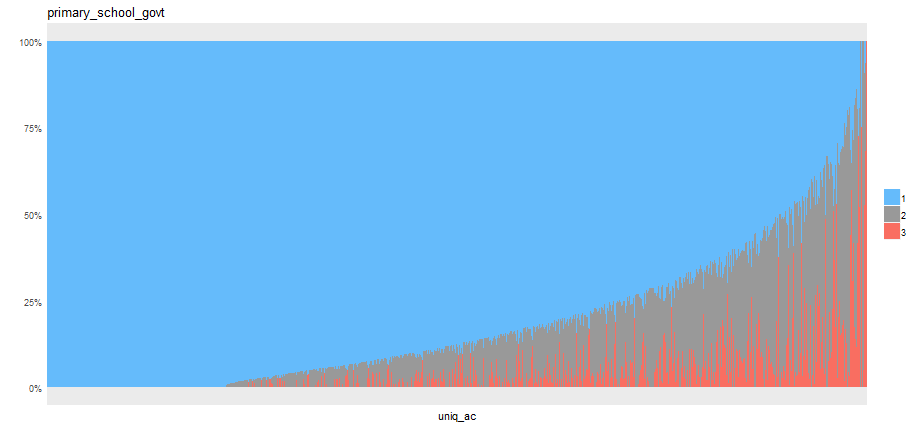


(e) private primary schools


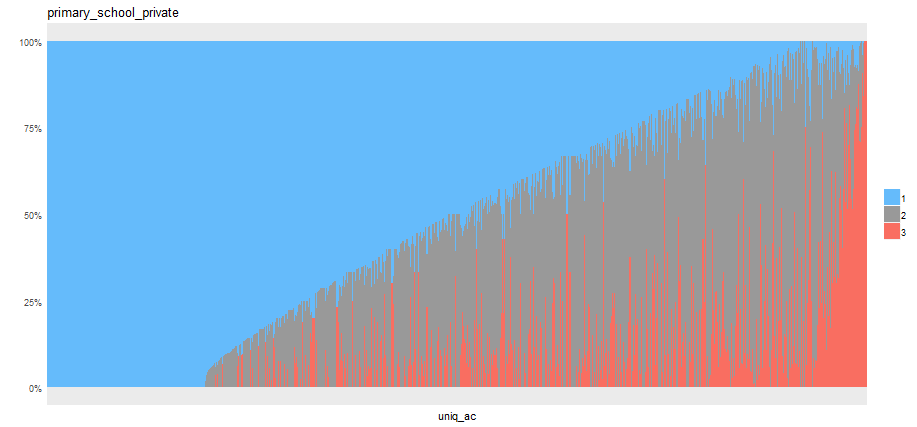


(f) government middle schools


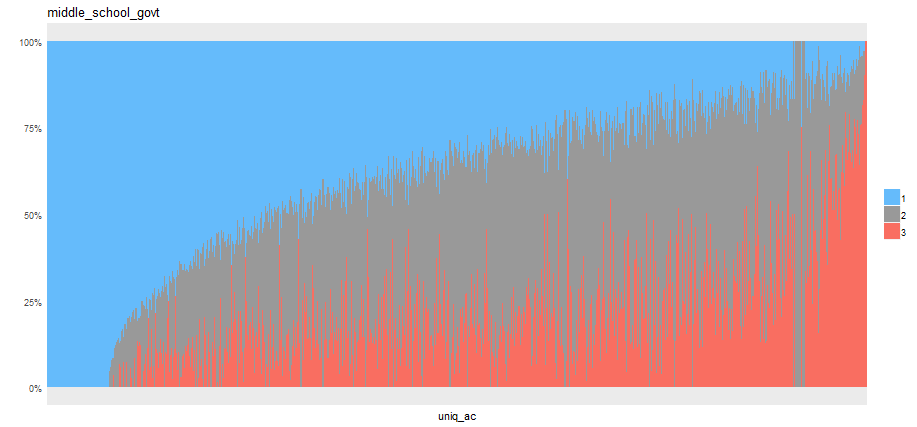


(g) private middle schools


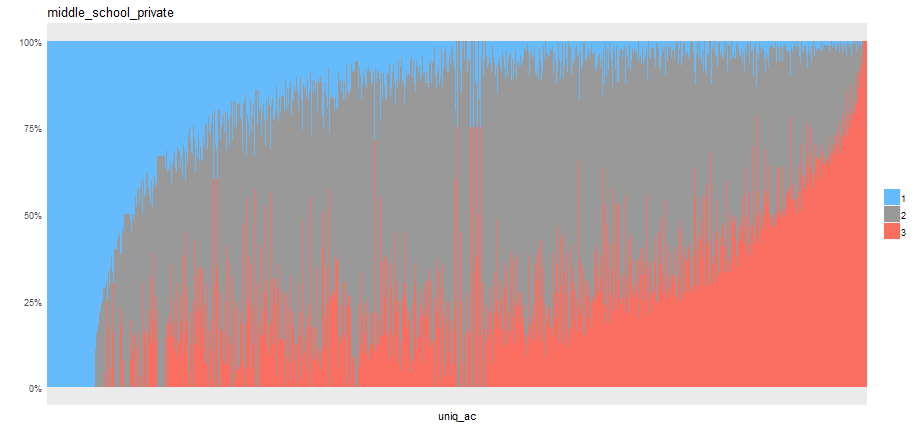


(h) government secondary schools


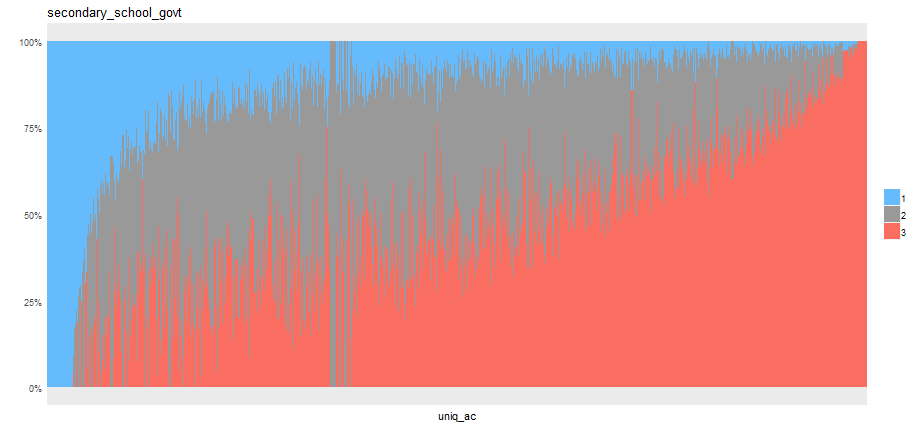


(i) private secondary schools


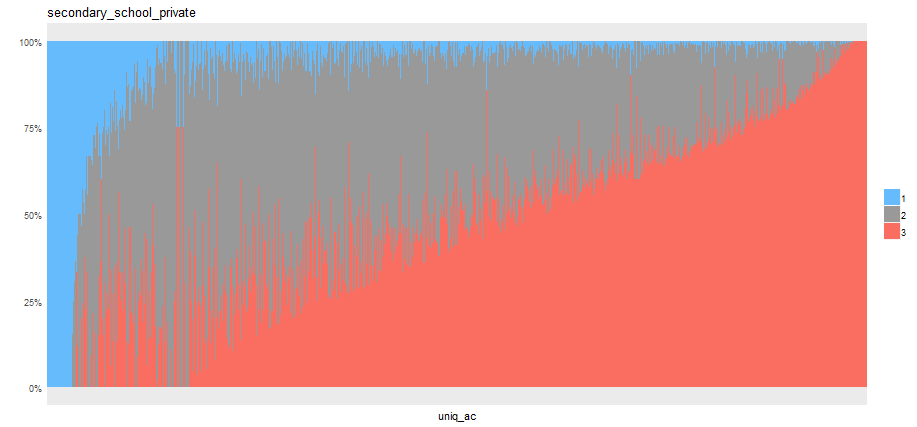


(j) government senior secondary schools


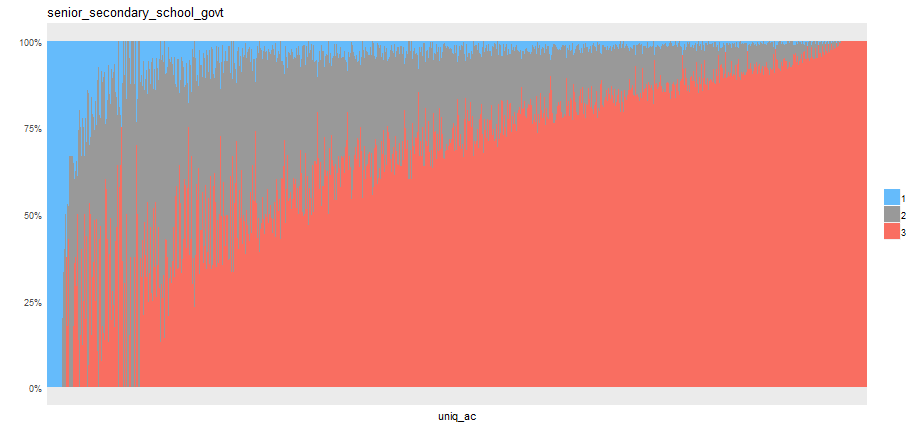


(k) private senior secondary schools

**
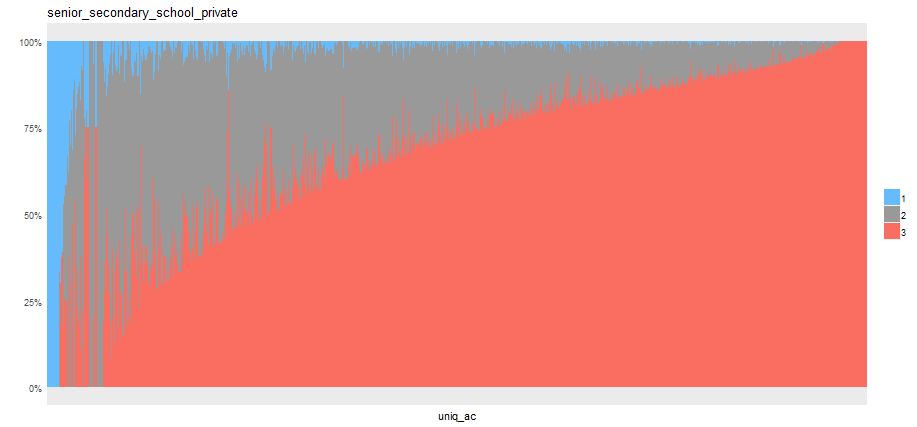
**

**Table S1a:** For each state, the interquartile range in InV% (percentage of villages in an AC where there is at least one facility is present in the village) across all ACs for 11 different educational infrastructures. G = government, P = private.

| **State** | **Anganwadi** | **Pre-prim. G** | **Pre-prim. P** | **Primary G** | **Primary P** | **Middle G** | **Middle P** | **Second. G** | **Second. P** | **Sen. Sec. G** | **Sen. Sec. P** |
| --- | --- | --- | --- | --- | --- | --- | --- | --- | --- | --- | --- |
| Andhra Pradesh | 2.94% | 0.00% | 26.38% | 3.60% | 20.00% | 24.69% | 32.95% | 22.98% | 12.59% | 4.03% | 4.24% |
| Arunachal Pradesh | 21.18% | 27.81% | 22.77% | 39.50% | 15.91% | 14.79% | 4.55% | 4.75% | 1.56% | 2.17% | 0.00% |
| Assam | 10.99% | 36.13% | 32.72% | 5.70% | 22.16% | 27.11% | 18.88% | 17.35% | 6.45% | 3.80% | 1.31% |
| Bihar | 10.79% | 0.00% | 36.32% | 12.05% | 43.73% | 19.61% | 11.76% | 8.46% | 2.85% | 3.94% | 1.56% |
| Chhattisgarh | 4.37% | 0.00% | 31.08% | 2.31% | 32.28% | 19.04% | 14.26% | 8.20% | 3.83% | 5.12% | 2.80% |
| Goa | 32.66% | 46.25% | 40.00% | 25.00% | 50.00% | 47.50% | 39.50% | 31.25% | 40.67% | 0.00% | 14.29% |
| Gujarat | 3.89% | 0.00% | 100.00% | 2.38% | 35.71% | 43.75% | 6.25% | 16.55% | 6.99% | 6.63% | 3.87% |
| Haryana | 1.43% | 0.00% | 15.24% | 5.26% | 13.34% | 22.03% | 40.85% | 34.22% | 34.19% | 23.79% | 19.51% |
| Himachal Pradesh | 15.89% | 8.26% | 18.31% | 24.15% | 17.89% | 12.97% | 6.59% | 8.06% | 2.97% | 5.16% | 2.23% |
| Jammu & Kashmir | 11.65% | 0.00% | 4.53% | 10.09% | 26.91% | 22.76% | 30.64% | 14.78% | 7.97% | 6.84% | 1.85% |
| Jharkhand | 34.43% | 0.00% | 28.74% | 15.21% | 38.64% | 14.69% | 7.98% | 4.61% | 3.82% | 1.66% | 1.45% |
| Karnataka | 18.50% | 21.03% | 53.03% | 11.17% | 49.99% | 35.15% | 36.62% | 18.21% | 11.16% | 4.67% | 3.94% |
| Kerala | 0.00% | 63.33% | 0.00% | 0.00% | 0.00% | 8.90% | 8.90% | 32.50% | 33.33% | 37.14% | 46.43% |
| Madhya Pradesh | 12.43% | 0.00% | 37.60% | 6.42% | 36.34% | 15.58% | 13.90% | 7.00% | 3.19% | 4.41% | 1.47% |
| Maharashtra | 3.67% | 2.54% | 50.00% | 4.11% | 53.85% | 26.11% | 18.02% | 20.06% | 6.72% | 8.83% | 3.01% |
| Manipur | 4.41% | 38.44% | 63.05% | 28.88% | 68.12% | 36.62% | 40.74% | 24.64% | 23.03% | 0.97% | 2.62% |
| Meghalaya | 23.31% | 15.21% | 39.95% | 20.50% | 48.52% | 20.38% | 28.42% | 8.87% | 9.73% | 1.70% | 2.08% |
| Mizoram | 15.39% | 42.91% | 12.50% | 3.23% | 15.71% | 16.52% | 61.11% | 34.21% | 22.33% | 0.00% | 7.18% |
| Nagaland | 17.65% | 0.00% | 44.97% | 14.71% | 50.00% | 20.73% | 20.01% | 13.55% | 14.29% | 0.00% | 0.00% |
| NCT of Delhi | 8.17% | 19.29% | 51.41% | 32.44% | 30.21% | 26.16% | 36.68% | 42.26% | 38.89% | 38.87% | 21.10% |
| Odisha | 14.70% | 0.00% | 26.19% | 14.07% | 34.94% | 20.37% | 10.76% | 12.34% | 4.89% | 2.48% | 1.32% |
| Puducherry | 0.00% | 1.92% | 0.00% | 0.00% | 0.00% | 31.59% | 41.67% | 28.90% | 21.15% | 23.66% | 7.14% |
| Punjab | 12.67% | 51.52% | 30.07% | 10.54% | 31.53% | 30.30% | 23.18% | 20.54% | 12.93% | 11.98% | 5.44% |
| Rajasthan | 3.32% | 0.00% | 30.09% | 20.41% | 30.33% | 28.00% | 26.10% | 18.21% | 17.36% | 8.17% | 7.15% |
| Sikkim | 8.90% | 31.70% | 21.07% | 15.67% | 20.24% | 16.67% | 33.33% | 16.87% | 0.00% | 15.45% | 0.00% |
| Tamil Nadu | 6.30% | 9.23% | 33.33% | 8.98% | 29.56% | 34.80% | 58.23% | 43.67% | 35.56% | 32.71% | 18.87% |
| Telangana | 4.10% | 0.00% | 16.35% | 1.79% | 10.83% | 24.44% | 32.47% | 24.70% | 14.49% | 3.25% | 2.85% |
| Tripura | 0.00% | 28.54% | 0.00% | 0.00% | 50.00% | 0.00% | 0.00% | 25.96% | 1.25% | 23.12% | 0.00% |
| Uttar Pradesh | 12.22% | 17.19% | 37.36% | 18.79% | 40.38% | 24.57% | 21.00% | 5.40% | 9.60% | 4.45% | 6.88% |
| Uttarakhand | 29.84% | 0.00% | 46.73% | 28.32% | 38.07% | 16.50% | 16.59% | 8.35% | 5.70% | 4.69% | 3.70% |
| West Bengal | 5.84% | 9.50% | 37.28% | 8.34% | 50.90% | 22.45% | 5.00% | 14.94% | 1.99% | 11.50% | 0.95% |

**Table S1b:** For each state, the interquartile range in OV≤5km% (percentage of villages in an AC where the facility is not present in the village *and* the nearest facility is within 5km) across all ACs for 11 different educational infrastructures. G = government, P = private.

| **State** | **Anganwadi** | **Pre-prim. G** | **Pre-prim. P** | **Primary G** | **Primary P** | **Middle G** | **Middle P** | **Second. G** | **Second. P** | **Sen. Sec. G** | **Sen. Sec. P** |
| --- | --- | --- | --- | --- | --- | --- | --- | --- | --- | --- | --- |
| Andhra Pradesh | 2.12% | 19.08% | 19.52% | 2.96% | 19.05% | 16.56% | 22.62% | 15.68% | 21.08% | 14.46% | 14.42% |
| Arunachal Pradesh | 8.25% | 18.93% | 27.18% | 18.25% | 28.71% | 19.08% | 26.82% | 18.38% | 19.90% | 9.25% | 9.94% |
| Assam | 8.47% | 17.02% | 27.56% | 4.49% | 19.83% | 19.94% | 20.08% | 15.77% | 15.77% | 16.12% | 16.61% |
| Bihar | 9.03% | 47.09% | 24.11% | 7.91% | 26.67% | 18.88% | 31.19% | 35.08% | 37.96% | 17.47% | 17.97% |
| Chhattisgarh | 2.91% | 23.13% | 23.48% | 2.06% | 24.56% | 17.62% | 17.84% | 18.83% | 19.81% | 17.60% | 19.58% |
| Goa | 14.56% | 36.01% | 39.38% | 25.00% | 50.00% | 50.96% | 44.05% | 68.86% | 47.14% | 62.80% | 52.38% |
| Gujarat | 1.30% | 4.74% | 50.00% | 1.34% | 16.67% | 31.25% | 50.00% | 13.97% | 19.20% | 14.15% | 13.49% |
| Haryana | 1.04% | 27.18% | 12.50% | 4.61% | 11.15% | 16.16% | 29.31% | 21.94% | 25.51% | 20.24% | 22.07% |
| Himachal Pradesh | 14.73% | 29.40% | 23.17% | 25.88% | 21.53% | 19.41% | 15.25% | 23.46% | 26.59% | 27.59% | 28.94% |
| Jammu & Kashmir | 4.82% | 33.33% | 2.82% | 9.37% | 23.23% | 20.36% | 31.19% | 34.35% | 33.44% | 30.53% | 31.19% |
| Jharkhand | 34.89% | 20.32% | 25.36% | 12.43% | 31.79% | 11.52% | 13.64% | 13.99% | 14.17% | 9.19% | 9.11% |
| Karnataka | 12.82% | 17.78% | 36.01% | 8.32% | 35.28% | 28.19% | 39.23% | 21.14% | 20.34% | 18.94% | 19.85% |
| Kerala | 0.00% | 46.43% | 0.00% | 0.00% | 0.00% | 0.00% | 0.00% | 20.00% | 22.22% | 33.33% | 33.33% |
| Madhya Pradesh | 11.68% | 29.95% | 33.33% | 5.82% | 30.76% | 13.48% | 13.88% | 12.34% | 15.76% | 11.37% | 12.28% |
| Maharashtra | 1.46% | 2.54% | 93.26% | 2.78% | 40.00% | 17.57% | 16.73% | 17.49% | 17.59% | 13.21% | 14.38% |
| Manipur | 3.21% | 33.33% | 49.61% | 21.69% | 50.00% | 32.86% | 34.41% | 43.85% | 37.71% | 50.33% | 46.75% |
| Meghalaya | 18.28% | 9.73% | 26.11% | 10.20% | 35.04% | 17.09% | 31.77% | 23.26% | 26.12% | 17.73% | 18.49% |
| Mizoram | 0.00% | 6.07% | 0.00% | 0.00% | 0.00% | 5.09% | 14.86% | 11.01% | 13.81% | 5.77% | 5.94% |
| Nagaland | 4.17% | 19.09% | 40.00% | 7.69% | 25.00% | 20.05% | 21.39% | 18.02% | 18.79% | 10.00% | 10.53% |
| NCT of Delhi | 8.17% | 18.03% | 53.33% | 33.33% | 33.33% | 29.16% | 36.09% | 60.00% | 51.76% | 61.82% | 40.87% |
| Odisha | 7.88% | 23.34% | 23.94% | 13.75% | 32.02% | 10.11% | 15.78% | 20.79% | 28.43% | 14.82% | 15.19% |
| Puducherry | 0.00% | 1.92% | 0.00% | 0.00% | 0.00% | 31.59% | 41.67% | 28.90% | 21.15% | 30.80% | 14.29% |
| Punjab | 7.72% | 32.18% | 28.26% | 9.80% | 32.04% | 26.75% | 20.78% | 23.53% | 20.73% | 20.17% | 18.30% |
| Rajasthan | 2.53% | 17.30% | 23.99% | 14.05% | 24.48% | 23.97% | 23.09% | 18.66% | 18.35% | 18.93% | 18.60% |
| Sikkim | 8.90% | 32.32% | 20.00% | 15.07% | 20.24% | 18.33% | 35.94% | 19.23% | 29.22% | 29.79% | 36.16% |
| Tamil Nadu | 5.57% | 6.78% | 25.81% | 6.72% | 21.15% | 32.64% | 52.85% | 26.37% | 30.91% | 25.80% | 27.72% |
| Telangana | 2.67% | 17.42% | 13.13% | 1.49% | 7.44% | 12.55% | 19.53% | 10.08% | 18.68% | 7.45% | 8.25% |
| Tripura | 0.00% | 42.32% | 0.00% | 0.00% | 33.33% | 0.00% | 0.00% | 15.95% | 35.21% | 26.03% | 34.96% |
| Uttar Pradesh | 7.63% | 29.32% | 35.55% | 17.49% | 38.80% | 23.48% | 20.66% | 23.10% | 21.96% | 25.05% | 22.89% |
| Uttarakhand | 27.55% | 16.73% | 42.60% | 28.25% | 41.55% | 21.85% | 20.13% | 17.45% | 20.30% | 19.71% | 20.85% |
| West Bengal | 5.64% | 6.11% | 36.15% | 7.95% | 47.18% | 21.28% | 17.28% | 15.39% | 18.51% | 20.66% | 24.84% |

**Table S1c:** For each state, the interquartile range in OV>5km% (percentage of villages in an AC where the facility is not present in the village *and* the nearest facility is over 5km away) across all ACs for 11 different educational infrastructures. G = government, P = private.

| **State** | **Anganwadi** | **Pre-prim. G** | **Pre-prim. P** | **Primary G** | **Primary P** | **Middle G** | **Middle P** | **Second. G** | **Second. P** | **Sen. Sec. G** | **Sen. Sec. P** |
| --- | --- | --- | --- | --- | --- | --- | --- | --- | --- | --- | --- |
| Andhra Pradesh | 0.00% | 19.08% | 0.00% | 0.00% | 0.00% | 12.48% | 24.24% | 22.31% | 28.34% | 15.58% | 15.69% |
| Arunachal Pradesh | 13.22% | 39.19% | 31.46% | 25.54% | 37.04% | 28.42% | 29.10% | 21.46% | 20.80% | 13.63% | 10.27% |
| Assam | 3.87% | 29.46% | 6.78% | 1.07% | 4.13% | 11.50% | 16.16% | 15.92% | 16.48% | 18.57% | 16.89% |
| Bihar | 3.01% | 47.09% | 15.25% | 4.74% | 19.68% | 17.11% | 29.08% | 36.89% | 40.56% | 19.28% | 19.02% |
| Chhattisgarh | 1.98% | 23.13% | 5.52% | 0.41% | 4.76% | 6.70% | 14.56% | 20.99% | 22.33% | 20.91% | 23.13% |
| Goa | 20.44% | 19.55% | 0.00% | 0.00% | 0.00% | 16.07% | 12.95% | 26.43% | 18.27% | 62.80% | 60.60% |
| Gujarat | 2.51% | 4.79% | 50.00% | 1.15% | 16.67% | 25.00% | 50.00% | 23.25% | 20.56% | 18.27% | 14.93% |
| Haryana | 0.00% | 27.18% | 3.45% | 2.04% | 3.85% | 8.11% | 12.32% | 17.70% | 21.27% | 19.94% | 25.88% |
| Himachal Pradesh | 3.73% | 39.29% | 4.98% | 2.61% | 6.07% | 11.03% | 16.07% | 22.51% | 26.56% | 27.66% | 31.01% |
| Jammu & Kashmir | 7.44% | 34.78% | 1.24% | 3.32% | 11.11% | 11.90% | 25.70% | 27.33% | 37.53% | 26.26% | 31.56% |
| Jharkhand | 0.00% | 20.32% | 7.86% | 2.03% | 8.43% | 7.78% | 10.99% | 18.37% | 15.79% | 9.74% | 10.39% |
| Karnataka | 3.03% | 23.04% | 20.24% | 4.46% | 17.67% | 8.62% | 17.64% | 15.49% | 19.26% | 18.64% | 18.60% |
| Kerala | 0.00% | 0.00% | 0.00% | 0.00% | 0.00% | 0.00% | 0.00% | 9.09% | 11.11% | 31.41% | 42.86% |
| Madhya Pradesh | 2.13% | 29.95% | 6.80% | 1.21% | 6.59% | 7.02% | 10.91% | 16.99% | 16.89% | 14.99% | 13.70% |
| Maharashtra | 0.80% | 0.00% | 40.00% | 0.63% | 10.00% | 11.07% | 19.81% | 14.23% | 14.84% | 15.45% | 15.12% |
| Manipur | 0.00% | 16.07% | 14.88% | 5.86% | 13.57% | 48.93% | 50.00% | 66.28% | 65.31% | 51.02% | 50.72% |
| Meghalaya | 12.17% | 6.06% | 11.32% | 6.21% | 14.39% | 21.09% | 19.40% | 27.01% | 24.92% | 20.48% | 18.83% |
| Mizoram | 15.67% | 39.24% | 0.00% | 0.00% | 0.00% | 12.92% | 41.79% | 33.04% | 23.31% | 8.85% | 10.56% |
| Nagaland | 14.29% | 19.09% | 28.57% | 8.33% | 22.62% | 25.00% | 29.87% | 24.84% | 22.04% | 12.50% | 11.11% |
| NCT of Delhi | 0.00% | 10.00% | 0.00% | 0.00% | 0.00% | 0.00% | 0.00% | 9.17% | 10.56% | 21.98% | 25.40% |
| Odisha | 7.11% | 23.34% | 10.05% | 1.82% | 7.80% | 16.88% | 17.08% | 23.43% | 32.00% | 17.13% | 15.91% |
| Puducherry | 0.00% | 0.00% | 0.00% | 0.00% | 0.00% | 0.00% | 0.00% | 0.00% | 0.00% | 3.57% | 7.14% |
| Punjab | 6.67% | 22.71% | 3.88% | 1.31% | 3.87% | 6.25% | 8.99% | 14.07% | 17.68% | 18.99% | 19.70% |
| Rajasthan | 1.14% | 17.30% | 8.22% | 5.67% | 8.44% | 9.83% | 10.93% | 14.50% | 18.37% | 21.33% | 22.83% |
| Sikkim | 0.00% | 12.95% | 0.00% | 0.00% | 0.00% | 10.83% | 20.00% | 18.96% | 31.01% | 37.52% | 36.16% |
| Tamil Nadu | 0.00% | 1.63% | 0.00% | 0.00% | 0.00% | 8.81% | 16.04% | 29.65% | 33.33% | 35.40% | 34.13% |
| Telangana | 1.22% | 17.42% | 0.00% | 0.00% | 0.00% | 13.06% | 25.35% | 23.38% | 24.28% | 9.60% | 9.08% |
| Tripura | 0.00% | 46.67% | 0.00% | 0.00% | 0.00% | 0.00% | 0.00% | 23.65% | 40.42% | 27.29% | 33.29% |
| Uttar Pradesh | 5.52% | 31.26% | 4.76% | 1.73% | 4.85% | 5.72% | 9.11% | 23.80% | 25.25% | 24.98% | 25.48% |
| Uttarakhand | 6.74% | 16.73% | 7.37% | 2.67% | 5.92% | 6.81% | 9.08% | 15.97% | 15.13% | 21.09% | 21.59% |
| West Bengal | 0.78% | 4.02% | 3.01% | 0.71% | 4.32% | 11.48% | 16.80% | 14.86% | 18.50% | 23.98% | 24.09% |
